# Embryonic thermal manipulation enhances splenic immunity and regulates inflammatory responses to *Escherichia coli* in broiler chickens

**DOI:** 10.3389/fvets.2026.1741919

**Published:** 2026-02-27

**Authors:** Mohammad Borhan Al-Zghoul, Rahmeh Okour, Daoud Alghizzawi, Khaled Musa Mohammad Saleh, Seif Hundam

**Affiliations:** 1 Department of Basic Medical Veterinary Sciences, Faculty of Veterinary Medicine, Jordan University of Science and Technology, Irbid, Jordan; 2 Department of Applied Biological Sciences, Faculty of Science and Art, Jordan University of Science and Technology, Irbid, Jordan; 3 Department of Basic Dental Sciences, Faculty of Dentistry, The Hashemite University, Zarqa, Jordan

**Keywords:** broiler, *Escherichia coli*, immune response, spleen immunity, thermal manipulation

## Abstract

**Introduction:**

Escherichia coli (*E. coli*) infections continue to pose a significant health and economic burden to the poultry industry, with increasing restrictions on antibiotic use underscoring the need for alternative strategies to improve host resilience. Thermal manipulation (TM) during embryogenesis has been proposed as an economical strategy to enhance thermotolerance, stress resilience, and immune functionality in broilers. This study aimed to investigate the effect of TM during embryonic development on the immune response of broiler chickens following an *E. coli* challenge.

**Methods:**

A total of 740 Ross broiler eggs were assigned to either a control incubation (37.8 °C, 56% RH) or TM (39 °C and 65% RH for 18 h daily, on embryonic days 10–18). After hatching, chicks were subdivided into saline- or *E. coli*-injected groups. Splenic expression of pro-inflammatory mediators (iNOS and TNF-α), signaling receptors (NF-κB, p65, TLR-2, and TLR-4), and immunoregulatory cytokine (TGF-β) was quantified by RT-qPCR. At the same time, serum levels of acute-phase proteins (α1-acid glycoprotein (AGP) and C3) and total circulating immunoglobulins (IgA, IgM, and IgY) were assessed by ELISA.

**Results:**

TM significantly modulated post-challenge immune responses, including downregulation of iNOS, TNF-α, NF-κB, TLR-2, and TLR-4, and upregulation of TGF-β. Notably, TM was associated with a stronger and more sustained circulating IgA response after infection. Additionally, TM lowered serum AGP levels under the *E. coli* challenge, which indicates reduced systemic inflammation.

**Discussion:**

These findings show that embryonic TM boosts both splenic and systemic immune regulation while reducing excessive inflammatory responses to bacterial challenges in broilers.

## 1 Introduction

The poultry industry offers high-quality animal protein and substantially contributes to economic stability by creating investment opportunities, employment, and income for smallholders. However, it encounters numerous challenges, notably those related to avian health hazards such as bacterial infections (1). Among these, *Escherichia coli* (*E. coli)* represents a primary concern, presenting considerable threats to the global poultry industry (2).

Historically, initiatives to address these infections primarily concentrated on employing antibiotics to enhance and support immune system functionality (3). The extended use of antibiotics has led to the emergence of bacterial resistance mechanisms, necessitating the prioritization of alternative remedies (4, 5). Probiotics and vaccinations have emerged as effective alternatives to antibiotics in poultry production (4, 5).

Concurrently, these treatments are expensive, exhibit significant variability in protective immunity, and may be unsuccessful due to problems related to vaccination or dietary supplements (6, 7). Thermal manipulation (TM) during broiler chicken embryogenesis is acknowledged as a significant alternative to these methods. TM demonstrated advantageous effects on tissue integrity, heat stress tolerance, and antioxidant activity in broiler chickens (8, 9). Furthermore, TM has been proposed to enhance hatchability, body weight, performance, meat quality, and overall favorable immunological response to heat stress in broiler chickens (9–12).

During TM, incubation temperature and humidity are cyclically adjusted at specific intervals of embryonic development in avian eggs (13). TM is a simple, cost-effective approach that has shown considerable promise in poultry production (14). Previous studies demonstrated its significant role in enhancing immune responses under both heat stress and microbial infections. In particular, TM has been shown to modulate splenic mRNA expression of cytokines, such as interleukins (IL-1β, IL-2, IL-4, IL-6, IL-8, IL-12, IL-15, IL-18), and interferon-gamma (IFN-γ)—during post-hatch heat stress and bacterial *E. coli* challenges (9, 15).

However, limited information is available regarding the influence of embryonic TM on immune responses to *E. coli* challenge. Therefore, the present study aimed to assess the effects of TM during embryogenesis, during *E. coli* challenge, on the expression levels of some pro-inflammatory mediators [inducible nitric oxide synthase (iNOS), tumor necrosis factor alpha (TNF-α)], signaling receptors [Nuclear factor kappa-light-chain-enhancer of activated B cells (NF-κB), p65, toll-like receptor 2 (TLR-2), toll-like receptor 4 (TLR-4), immunoregulatory cytokine (transforming growth factor beta (TGF-β)], acute-phase proteins [α1-acid glycoprotein (AGP) and complement component 3 (C3)], and total circulating immunoglobulins (IgA, IgM, and IgY). Unlike previous studies that focused on individual immune markers or specific stress conditions, this study provides a comprehensive and integrated evaluation of both splenic and systemic immune responses to *E. coli* infection, thereby highlighting the novelty of the present work.

## 2 Materials and methods

The animal study was reviewed and approved by the Animal Care and Use Committee at Jordan University of Science and Technology (JUST) under approval number (475/2020). All experimental procedures were conducted in accordance with institutional guidelines, national regulations for animal welfare, and the ARRIVE guidelines for reporting animal research.

### 2.1 Study period and location

The research was conducted between June 5, 2020, and June 30, 2020, at the Animal House Unit, located within the Faculty of Veterinary Medicine at Jordan University of Science and Technology (JUST) in Irbid, Jordan.

### 2.2 Study population and incubation

A total of 740 fertile Ross 308 broiler eggs were obtained from a single breeder flock (36 weeks old) from authorized suppliers in Irbid, Jordan, and underwent a thorough inspection to exclude any that were damaged or exhibited abnormalities. Eggs within an average weight range of 63 ± 2 grams were selected, while those significantly above (>70 grams) or below (< 55 grams) the average were discarded. The selected eggs were distributed equally across four identical incubators (Masalles, Barcelona, Spain; model Mod.25-IPDS) to represent two independent biological replicates per treatment (two incubators for the control and two for thermal manipulation; *n* = 185 eggs per incubator). Two incubators per treatment were used, and the incubator was considered the experimental unit for incubation-related effects to avoid pseudo-replication.

The control group was incubated at a constant temperature of 37.8 °C and 56% relative humidity (RH) throughout embryonic development. In contrast, the TM group was incubated under standard conditions, except during embryonic days (ED) 10–18, when the temperature was elevated to 39 °C and the relative humidity (RH) was increased to 65% for 18 h each day. From the first day of incubation, all eggs were kept under tightly regulated conditions, including a temperature of 37.8 °C, 56% RH, and automatic hourly turning. On the 7th day, a candling process was conducted to evaluate the viability of the eggs. Consequently, infertile eggs and those containing non-viable embryos were removed. During the TM period, eggs remained in the same incubators, and incubation proceeded continuously while temperature and RH were adjusted to the target settings and allowed to reach and stabilize at these conditions. The incubation room temperature was maintained at 25 °C to minimize environmental fluctuations and prevent embryonic thermal shock.

### 2.3 Hatching management and rearing

Once hatched and dried, chicks were relocated to the Animal House Unit at JUST to commence the field experiment and randomly allocated into cage pens (8 birds per pen), with the pen considered the experimental unit. The initial room temperature was maintained at 35 °C ± 1 °C for the first week, followed by a gradual decrease in temperature. From day 20 to day 25 post-hatching, the temperature stabilized at 22 °C.

Chickens were fed basal diets as recommended by the National Research Council to meet the standard nutrient requirements of birds (16). All birds had free access (ad libitum) to feed and fresh water. Two feeding phases of corn-soybean meal-based rations were utilized: Starter (d 1–10), Grower (d 11–25), with levels of metabolizable energy and crude protein of 3,000 and 3,100 kcal/kg, and 23% and 20%, respectively. The experiment was terminated on day 25, and all broiler chickens were humanely euthanized by cervical dislocation. This procedure was approved by the Animal Care and Use Committee at Jordan University of Science and Technology (JUST).

### 2.4 *E. coli* inoculum preparation

*E. coli* serotype 078 was used to prepare the inoculum, which the JUST microbiology lab supplied. The bacterial strain was grown overnight in Muller–Hinton broth at 37 °C with continuous shaking. A 2 mL aliquot of the culture was centrifuged at 5,000 × g for 5 min at 4 °C. The resulting pellet was washed twice with 2 mL of 1 × sterile phosphate-buffered saline (PBS) and resuspended in the buffer, followed by another centrifugation step under the same conditions. It was then resuspended in 2 mL of 0.9% normal saline (NS). The optical density was measured at 600 nm (OD600) using a spectrophotometer and adjusted to the required concentration using a previously established OD600–CFU standard curve. Appropriate serial dilutions were prepared in NS to obtain the target inoculum concentration.

### 2.5 Bacterial challenge

Upon reaching 20 days of age, broilers underwent an *E. coli* challenge. Both the control and thermal manipulation groups were divided into two subgroups: a normal saline (NS) subgroup (*n* = 100 per group) and an *E. coli* subgroup (*n* = 100 per group). Birds in the NS subgroup received an intraperitoneal injection of 0.5 mL of 0.9% normal saline (NS), while those in the *E. coli* subgroup were injected intraperitoneally with 0.5 mL of E. coli suspension containing 1.5 × 105 colony-forming units (CFU)/mL. Subsequently, the broilers were transferred to an experimental room maintained at thermoneutral conditions (24 °C ± 1.0 °C).

### 2.6 RNA isolation and cDNA synthesis

Spleen tissues were collected from 40 broilers on post-injection days 1, 3, and 5 after challenge with *E. coli* or NS. To ensure RNA integrity, the samples were immediately snap-frozen in liquid nitrogen after collection. The tissues were stored at −80 °C in TRI Reagent solution tubes (Zymo Research Co., CA, USA). Homogenization of the tissues was performed using a Bead Ruptor Elite-Bead Mill Homogenizer (OMNI International, Kennesaw, GA, USA). Total RNA was extracted from splenic tissues using the Direct-Zol RNA MiniPrep kit (Zymo Research Co., CA, USA) with TRI Reagent (Zymo Research Co., CA, USA). The quantity and quality of the extracted RNA were assessed using a Qubit 4 Fluorometer (Thermo Fisher Scientific, MA, USA), a Biotek PowerWave XS2 Spectrophotometer (BioTek Instruments, Inc., Winooski, VT, USA), and 1% agarose gel electrophoresis. For cDNA synthesis, 500 ng of RNA per sample was reverse transcribed using the PrimeScript RT Master Mix (Perfect Real Time) (TaKaRa Bio Inc. Kusatsu, Shiga, Japan).

### 2.7 Real-time quantitative polymerase chain reaction (qPCR)

Relative quantitative qPCR was conducted using Blastaq Green qPCR Master Mix (Applied Biological Materials Inc., Richmond, Canada) on a Rotor-Gene Q MDx 5 Plex instrument (Qiagen, Hilden, Germany). Each 20 μL reaction mix included 10 μL of master mix, 2 μL of forward and reverse primers (2 pmol each), 2 μL of cDNA template, and 4 μL of nuclease-free water. The thermal cycling protocol consisted of an initial step at 50 °C for 2 min, followed by 95 °C for 15 min, and 40 cycles of 95 °C for 10 s, 57 °C for 30 s, and 72 °C for 10 s, concluding with a final melting step at 95 °C for 20 s. Duplicate reactions were performed for each cDNA sample, and fluorescence detection allowed for automatic relative quantification. Internal control genes (β-actin, 28S rRNA, and glyceraldehyde-3-phosphate dehydrogenase) were used for normalizing gene expression. Amplification specificity was verified through melting curve analysis. cDNA sequences were obtained from the NCBI Nucleotide database (https://www.ncbi.nlm.nih.gov/nucleotide/), and primers were designed using IDT PrimerQuest software (http://eu.idtdna.com/PrimerQuest/Home/Index). Primer sequences are presented in Table 1.

**Table 1:** Primer sequences used in real-time qPCR analysis.

**The gene**	**Sequence (5^′^-3^′^)**	**Annealing temperature (°C)**
28S rRNA	F: CCTGAATCCCGAGGTTAACTATT R: GAGGTGCGGCTTATCATCTATC	60
β-Actin	F: ACCGCAAATGCTTCTAAACC R: ATAAAGCCATGCCAATCTCG	60
GAPDH	F: TTGTCTCCTGTGACTTCAATGGTG R: ACGGTTGCTGTATCCAAACTCAT	60
iNOS	F: CCTGGAGGTCCTGGAAGAGT R: CCTGGGTTTCAGAAGTGGC	60
TNF-α	F: TGTGTATGTGCAGCAACCCGTAGT R: GCATTGCAATTTGGACAGAAGT	60
NF-κB	F: GCACAACGCCTCTTCACATA R: GGCTCAAAGTTCTCAACGTG	60
TLR2	F: GCCACAGACATTCCTAAC R: ATTCACAGGAGCAGATAAAG	60
TLR-4	F: GACAGCTTCTCAGCAGGCAAT R: CGGTTGGTGGACCTGAATCT	60
NF-kB p65	F: CATTGCCAGCATGGCTACTA R: TCCAGTTCCCGTTTCTTCAC	60
TGF-β	F: GGGTGTCCCATACCATTTAGAG R: CCCTTTAACGCAGAGGGATT	60

### 2.8 Enzyme-linked immunosorbent assay (ELISA)

On post-injection days 1, 3, and 5, blood samples were collected via the wing vein from the same randomly selected birds used for qPCR sampling, using sterile syringes, and were transferred into plain tubes (without anticoagulant). Samples were allowed to clot at room temperature and then centrifuged at 3,000 × g for 10 min to separate serum. The harvested serum was transferred to sterile microtubes and stored at −20 °C until analysis. The concentration of serum acute-phase proteins (AGP and total C3) and total circulating immunoglobulins (IgA, IgM, and IgY) was determined using a double antibody-sandwich ELISA detection method, which forms immunological complexes between an antigen and two layers of antibodies conjugated to a specific enzyme. Each set of commercial kits (Wuhan Fine Biotech Co., Ltd., Wuhan, China) included instructions that were followed during the procedure. Absorbance was recorded 10 min after the reaction was stopped, using a Biotek PowerWave XS2 Spectrophotometer (BioTek Instruments, Inc., Winooski, VT, USA).

### 2.9 Statistical analysis

Statistical analyses were carried out using IBM SPSS Statistics 27.0 (IBM Software, Chicago, IL, USA). All analyses were performed using the pen as the experimental unit, as all measurements were collected during the post-hatch period. At each sampling time point (days 1, 3, and 5 post-injection), 40 broilers were sampled per treatment group per day (i.e., 40 birds per experimental group). Birds were sampled from multiple pens, and individual measurements obtained from birds within the same pen were averaged to generate a single mean value per pen. These pen-level means were used as independent biological replicates for the two-way ANOVA. A two-way ANOVA was performed with embryonic TM (control vs. TM) and post-hatch treatment (normal saline vs. *E. coli*) as fixed factors, including their interaction (TM × treatment). Data for mRNA expression and serum levels were presented as means ± standard deviation. A two-way ANOVA analysis was used to evaluate differences in mRNA expression and serological (ELISA) levels between the control and TM groups at various time points post-NS and *E. coli* injections. For gene expression analysis, Ct values were normalized against reference genes (β-Actin, 28S rRNA, and GAPDH), and relative expression was calculated using the 2–^ΔΔ*Ct*^ method. When a significant difference was detected, multiple means comparisons were performed using Tukey's honestly significant difference (HSD) *post-hoc* test. Differences were considered statistically significant at a *p-value* < 0.05.

## 3 Results and discussion

This study aimed to evaluate the impact of TM during embryogenesis, concurrent with an *E. coli* challenge, on splenic pro-inflammatory mediators iNOS, TNF-α, NF-κB, p65, TLR-2, TLR-4, TGF-β, serum levels of AGP, C3, IgM, and IgY. The TM-treated groups were incubated under standard conditions, except on ED 10–18, during which they were incubated at 39 °C air temperature and 65% RH for 18 h daily.

### 3.1 Effects of thermal manipulation (TM) and post-hatch *E. coli* challenge on broiler chickens' iNOS splenic mRNA expression levels

**Figure 1:**
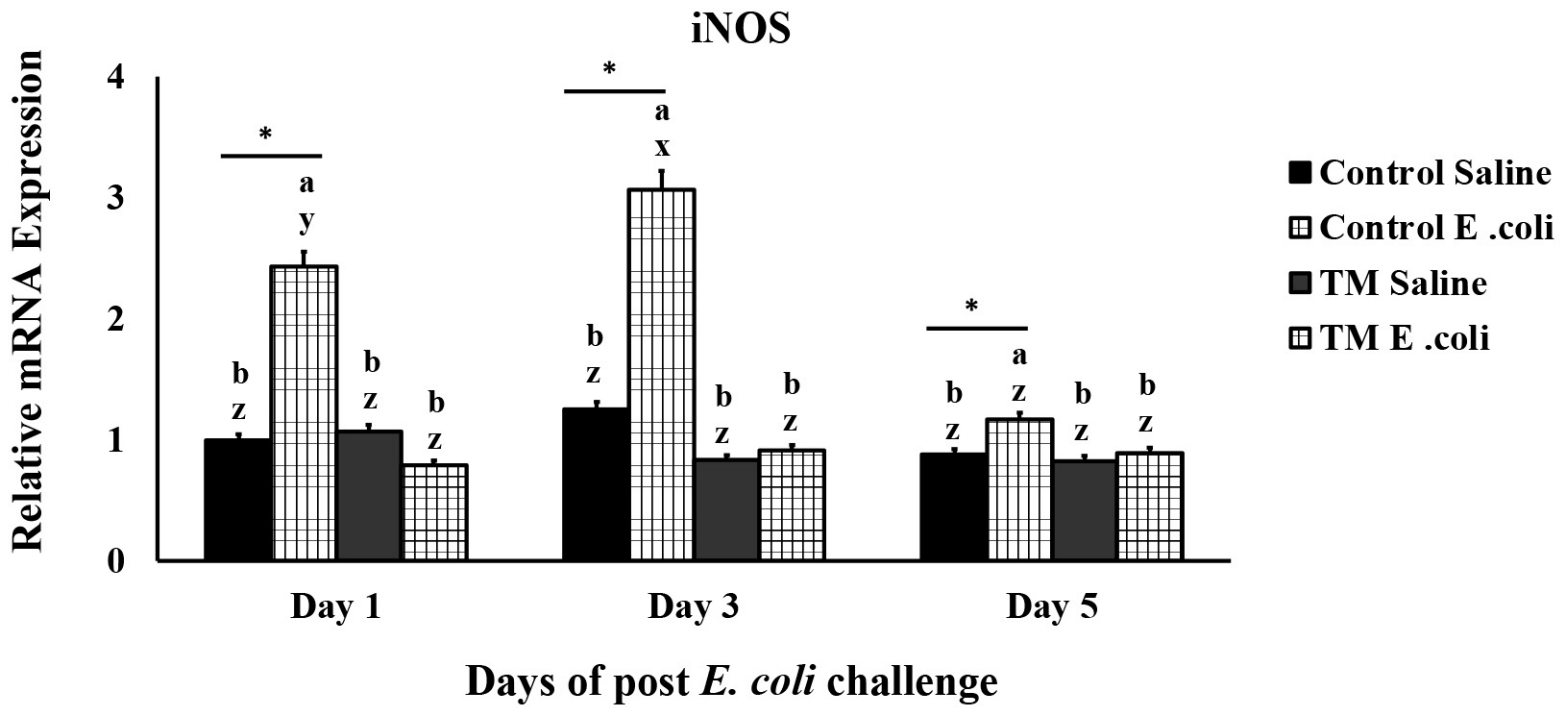
Effects of thermal manipulation (TM) during embryogenesis on the splenic mRNA levels of iNOS in broiler chickens following *Escherichia coli* challenge. Different letters (a–b) indicate significant differences among treatments, while different letters (x–z) indicate significant differences among time points within the same treatment (*p* < 0.05). Error bars represent mean ± standard deviation (SD). *,*Within the same day, the mean of the TM group is significantly different from the mean of the control (*p* < 0.05).

As illustrated in Figure 1, the control group treated with *E. coli* demonstrated a higher level of iNOS expression compared to the saline-treated control group on the first day (*p* < 0.01). iNOS expression further increased from day 1 to day 3 (*p* < 0.05) and subsequently declined from day 3 to day 5 (*p* < 0.001). Within the TM-treated group, both saline-injected and *E. coli*-infected subsets exhibited similar iNOS expression levels starting from the first day (*p* > 0.05). Upon the *E. coli* challenge, the TM group consistently displayed lower levels of iNOS expression than the control group throughout the post-infection period (*p* < 0.05). Notably, in the TM group, the iNOS expression levels for both saline-injected and *E. coli*-infected subsets remained nearly identical throughout the observation period (*p* > 0.05).

Inducible nitric oxide synthase (iNOS) is an enzyme responsible for producing nitric oxide (NO), synthesized in response to cytokines and other immunological stimuli (17, 18). While NO is an important effector molecule produced downstream of inflammatory signaling cascades following pathogen recognition, its overexpression may lead to cell death by apoptosis, making it a valuable marker for inflammation (19). Uncontrolled iNOS activity is linked to the advancement of chronic inflammatory diseases, neurodegeneration, and specific cancers, highlighting the necessity for stringent regulation of its expression in cells (20, 21).

Upregulation of the iNOS gene has been documented in broiler chickens exposed to stress. A previous study detected higher iNOS expression levels in chickens treated with LPS from various *E. coli* and *S. enteritica* during heat stress compared to controls (22). Similarly, *Salmonella* Typhimurium infection elevates iNOS levels and NO production in chickens as reported by Singh et al. (23). In contrast, Chen et al. (24) found that 5-aminolevulinic acid (5-ALA) supplementation reduced iNOS expression, contributing to a more balanced immune response. Additionally, maintaining low iNOS expression in the vascular endothelium has been linked to blood vessel stability, whereas excessive iNOS activity during infection may cause cytotoxic effects (25).

These results showed that TM influenced the iNOS pathway in broiler chickens exposed to *E. coli*. Although nitric oxide concentrations were not directly measured in this study, the reduction in iNOS gene expression observed in the TM-treated group compared with controls indicates a possible downregulation of NO synthesis, a mechanism previously linked to decreased tissue injury during infection (26). Such regulation is beneficial in controlling excessive inflammation, which may compromise the host's health (27). Furthermore, the lack of marked variation in iNOS expression between saline-injected and *E. coli*-infected groups within the TM group implies that it may contribute to maintaining a balanced immune response under pathogenic stress. These results support the potential of TM as a practical intervention for enhancing poultry health and resilience to bacterial challenges.

### 3.2 Effects of thermal manipulation (TM) and post-hatch *E. coli* challenge on broiler chickens' TNF-α splenic mRNA expression levels

**Figure 2:**
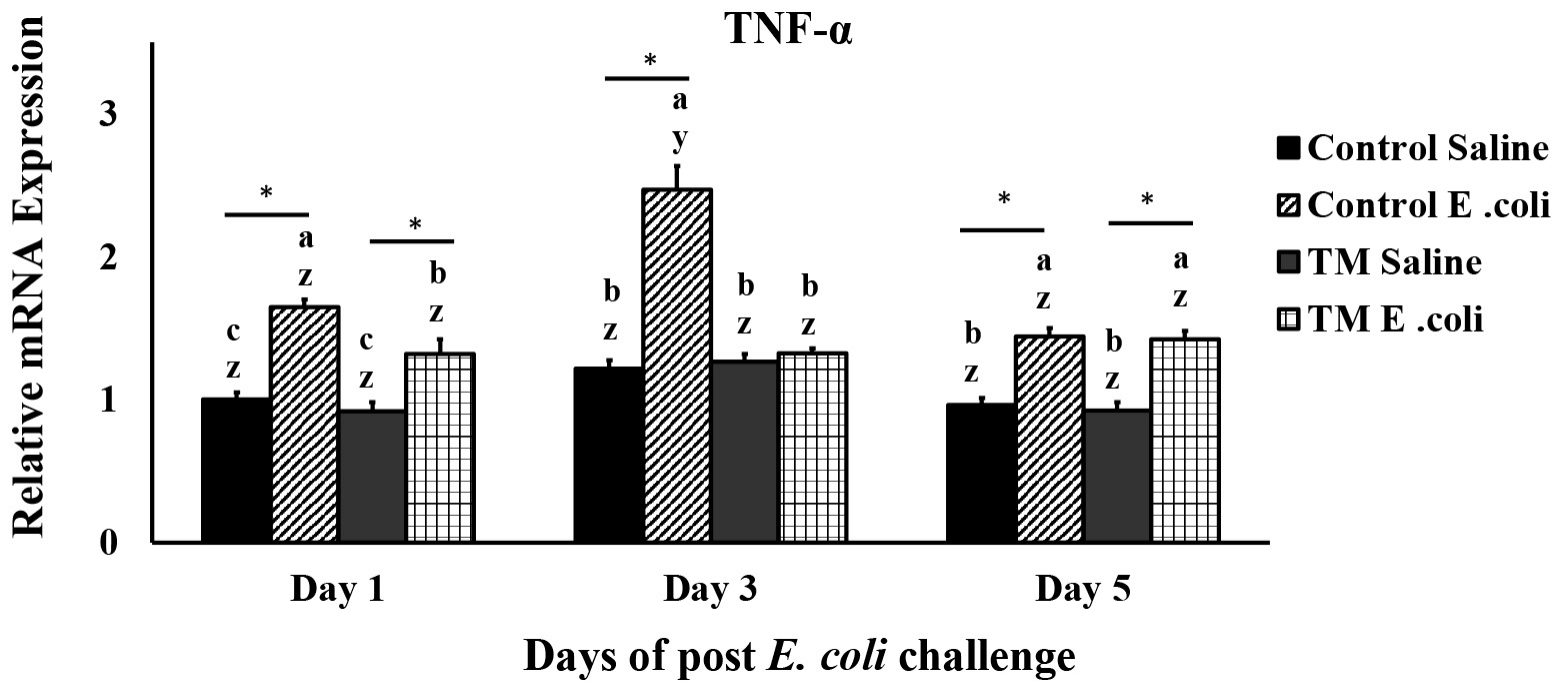
The effects of thermal manipulation (TM) during embryogenesis on the splenic mRNA expression levels of TNF-α in broiler chickens following an *Escherichia coli* challenge are examined. Different letters (a–c) indicate significant differences among treatments, while different letters (x–y) indicate significant differences among time points within the same treatment (*p* < 0.05) *,*Within the same day, the mean of the TM group is significantly different from the mean of the control (*p* < 0.05). Error bars represent mean ± standard deviation (SD).

TNF-α levels were similar between the saline-injected control and saline-injected TM group throughout the study (Figure 2, *p* > 0.05). After *E. coli* infection, the control group showed a significant increase in TNF-α on days 1 and 3 compared to both the saline-injected controls and TM-treated groups (*p* < 0.05). On the fifth day, the level of TNF-α expression was similar in both *E. coli*-infected groups as well as in both saline-injected groups (*p* > 0.05).

Tumor Necrosis Factor-alpha (TNF-α) is a pro-inflammatory cytokine, mainly produced by immune cells like macrophages. It plays an important role in the immune system by promoting inflammation, attracting immune cells to the site of infection, and regulating other cytokines and signaling pathways (28, 29). However, overexpression of TNF-α is linked to several inflammatory diseases like rheumatoid arthritis, inflammatory bowel disease, and psoriasis (30).

Elevated levels of TNF-α have been observed in *E. coli*-infected chickens exposed to heat stress (31). Similarly, Lu et al. (32) reported increased TNF-α expression in chickens infected with *Eimeria tenella* at various stages of infection. Notably, high TNF-α levels were associated with damage to intestinal epithelial cells, facilitating the invasion of commensal bacteria and triggering both local and systemic inflammatory responses (32). Additionally, Abbas et al. (33) found that the resulting inflammation from *E. coli* infection was significantly associated with increased TNF-α expression levels (33). Conversely, an improved immune response was reported after supplementation with a combination of oil blend, L-Arg, and Vit E, which reduced TNF-α expression levels (34). Zhang et al. (35) also reported decreased splenic TNF-α expression as a positive effect of resveratrol supplementation in broiler chickens.

In this study, the *E. coli*-infected control group exhibited significantly higher TNF-α mRNA expression levels compared to the TM-treated group. The observed reduction in TNF-α expression in the TM-treated group may enhance innate immune responses against *E. coli* infection, thereby contributing to a reduced pathogenic load.

### 3.3 Effects of TM and post-hatch *E. coli* challenge on broiler chickens' NF-κB, p65, TLR2, and TLR-4 splenic mRNA expression levels

**Figure 3:**
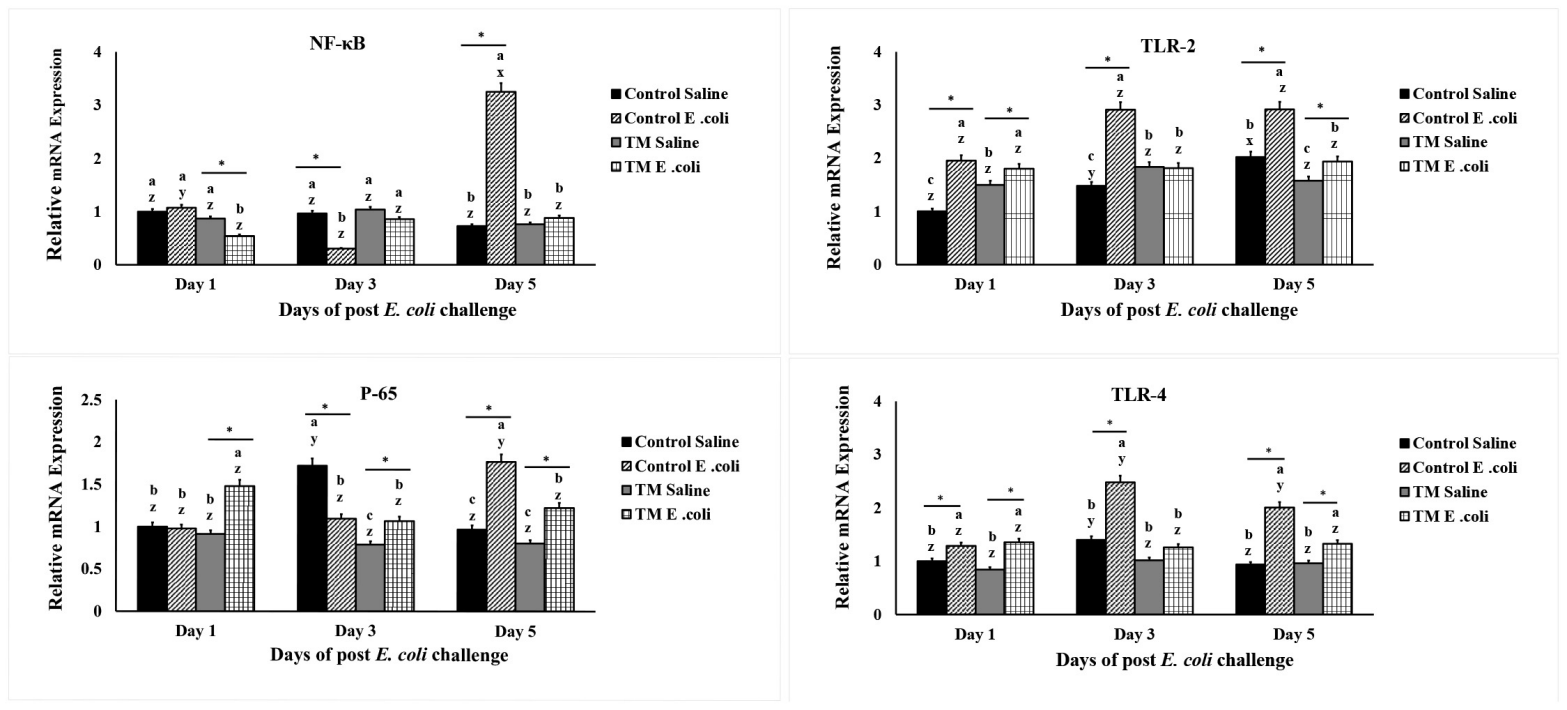
The effects of thermal manipulation (TM) during embryogenesis on the splenic mRNA levels of NF-κB, p65, TLR-2, and TLR-4 in broiler chickens following *Escherichia coli* challenge are examined. Means with different superscripts are significantly different (*p* < 0.05). *,*Within the same day, the mean of the TM group is significantly different from the mean of the control (*p* < 0.05). Error bars represent mean ± standard deviation (SD).

As shown in Figure 3, thermal manipulation (TM) influenced the splenic mRNA expression of genes involved in signaling receptor pathways following *E. coli* challenge. Specifically, NF-κB expression was significantly reduced in the TM *E. coli* group compared to the *E. coli* control at days 1 and 5 post-infection, while on day 3, TM *E. coli* birds showed higher levels than the *E. coli* control (*p* < 0.05).

Similarly, p65 expression in the TM *E. coli* group was lower on day 5 but higher on day 1 compared to the *E. coli*-challenged control group (*p* < 0.05). Regarding the splenic expressions of TLR-2 and TLR-4, *E. coli* infection markedly upregulated TLR-2 and TLR-4 expression across days 1, 3, and 5 (*p* < 0.05). In contrast, TM significantly suppressed this upregulation, resulting in lower expression levels than those observed in the *E. coli* challenged control group at days 3 and 5 post-infection (*p* < 0.05).

NF-κB is a transcription factor involved in regulating immune responses, inflammation, cell survival, and proliferation (36). Upon inflammation or pathogen infection, inhibitory proteins are phosphorylated, and NF-κB is released to reach the nucleus (37). Thereby, it can bind DNA and promote the expression of several pro-inflammatory target genes, including TNF-α, IL-6, IL-1, and IL-8 (36, 38). The p65 subunit, in particular, plays a crucial role in mediating the transcriptional activity of NF-κB, often through interaction with coactivators and regulation of chromatin remodeling (37).

Toll-like receptors (TLRs) are a group of protein receptors that recognize conserved microbial components, playing an important role in initiating innate immune response (39). TLR-2 is known for its ability to recognize components of Gram-positive bacteria, while TLR-4 recognizes Gram-negative bacteria by detecting lipopolysaccharide (LPS), a significant component of its outer membrane (40, 41). Both TLR-2 and TLR-4 trigger signaling cascades, which activate NF-κB and produce pro-inflammatory cytokines such as TNF-α and IL-6 when they are activated (42). Early immune responses and the development of the adaptive immune system depend on these receptors (41, 43).

The activation of NF-κB has been closely linked to the development of various inflammatory diseases (36). In poultry, NF-κB upregulation can lead to systemic inflammation, increased production of pro-inflammatory cytokines as a result of elevated oxidative stress levels (44). To counter these effects, nutritional supplements have been proposed as modulators that reduce NF-κB expression and help maintain redox homeostasis (44). During bacterial infections such as those caused by *E. coli*, both TLR-2 and TLR-4 are significantly upregulated, contributing to an excessive immune response (44). Similarly, infection with *Clostridium perfringens* has been shown to increase splenic TLR-2 and TLR-4 expression in poultry, indicating their key role in mediating pathogen recognition and inflammation (45). Conversely, downregulation of TLR-2, TLR-4, and NF-κB has been observed as a marker of immune system recovery in animals undergoing exercise therapy following cardiac ischemia (46).

In this study, the expression of NF-κB, p65, TLR-2, and TLR-4 was generally lower in the TM *E. coli*-infected group compared to the *E. coli* control. Since these genes are central mediators of inflammation and immune signaling, their suppression suggests that TM may attenuate excessive inflammatory responses induced by *E. coli*. By dampening immune overactivation, TM could help protect tissues from immunopathological damage and promote a more balanced host response to bacterial challenge.

### 3.4 Effects of the TM and post-hatch *E. coli* challenge on broiler chickens' TGF-β4 mRNA expression

**Figure 4:**
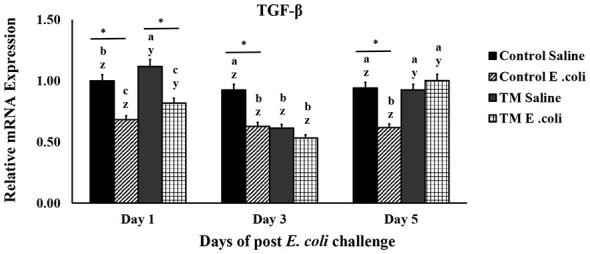
Effects of thermal manipulation (TM) during embryogenesis on the spleen levels of Transforming Growth Factor-β4 (TGF) in broiler chickens after *Escherichia coli* challenge. Different letters (a–c) indicate significant differences among treatments (*p* < 0.05). *,*Within the same day, the mean of the TM group is significantly different from the mean of the control (*p* < 0.05). Error bars represent mean ± standard deviation (SD).

Figure 4 illustrates the impact of TM and *E. coli* challenge on TGF-β4 levels in broiler chickens. The control *E. coli* challenge group consistently maintained lower TGF-β4 levels compared with the corresponding saline group throughout the study (*p* < 0.05). In contrast, the TM *E. coli* challenged groups exhibited higher TGF-β4 levels on day 5 compared to the control *E. coli* challenged group (*p* < 0.05).

TGF-β is a multifunctional cytokine that regulates cell growth, immune response, and tissue repair (47). It plays a central role in the development of T-lymphocytes and suppresses excessive immune activation, thereby maintaining immune tolerance (48). Moreover, TGF-β promotes extracellular matrix production to support wound healing, while its dysregulation contributes to pathological fibrosis (49). In cancer, TGF-β acts as a tumor suppressor in early stages but promotes invasion and immune evasion in advanced disease (50).

A previous study found that higher TGF-β1 expression was linked to better anti-inflammatory responses in broiler chickens (34). On the other hand, heat stress was shown to lower TGF-β levels, but interestingly, supplementing the diet with copper oxide nanoparticles helped bring those levels back up, which in turn improved the chickens' anti-inflammatory response (51). Consistent with our findings, previous research during *Eimeria tenella* infection observed an increase in splenic TGF-β4 expression during the later stages of the disease, suggesting a critical role in tissue repair and immune homeostasis (52). This aligns with the report by Karaffová et al. (53), who noted that TGF-β4 expression is predominantly enhanced during the late phase of *Salmonella* Enteritidis infection. Given that TGF-β4 is produced in intestinal villi, it likely plays a functional role in modulating the regeneration and repair of intestinal architecture following inflammatory damage.

In the present study, TGF-β4 levels were higher in the TM group compared with the *E. coli*–challenged control group on day 5. Because TGF-β4 is an important immunoregulatory cytokine involved in limiting excessive inflammation and promoting anti-inflammatory responses, its elevation in TM birds may reflect enhanced immune regulation during the later phase of the response to *E. coli* challenge. This pattern is consistent with the possibility of a faster resolution of inflammation in TM-treated birds; however, this interpretation should be considered cautiously and would require confirmation using additional inflammatory and clinical outcome measurements.

### 3.5 Effects of TM and post-hatch *E. coli* challenge on broiler chickens' AGP and C3 serum levels

**Figure 5:**
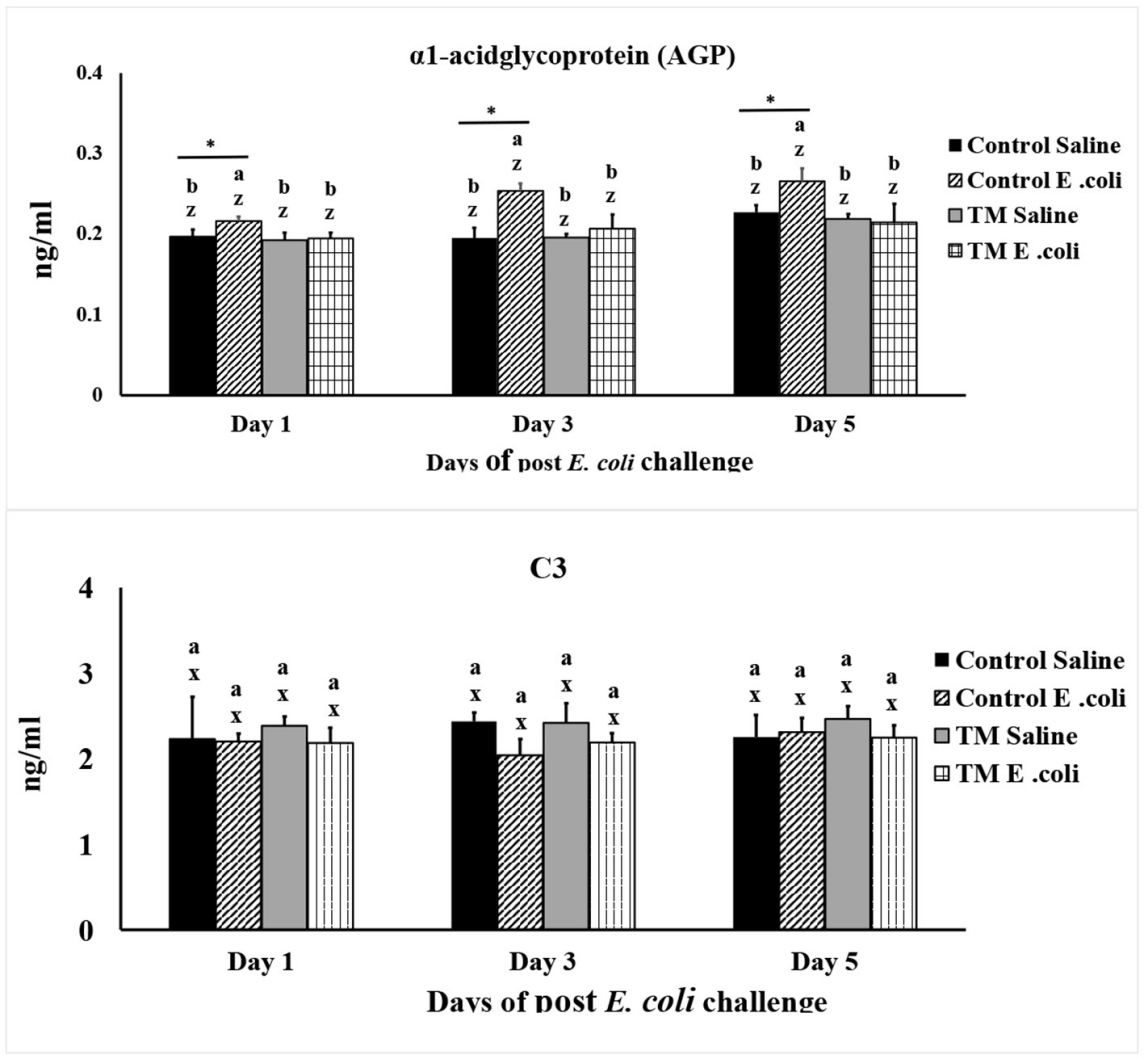
Effects of thermal manipulation (TM) during embryogenesis on the blood levels of α1-Acid Glycoprotein (AGP) and C3 in broiler chickens after *Escherichia coli* challenge. Means with different superscripts are significantly different (*p* < 0.05). *,*Within the same day, the mean of the TM group is significantly different compared to the mean of the control (*p* < 0.05). Error bars represent mean ± standard deviation (SD).

Figure 5 shows the impact of TM and *E. coli* challenge on blood levels of α1-acid glycoprotein (AGP) and C3 in broiler chickens. The highest levels of AGP were consistently found in the *E. coli*-infected control group throughout the study (*p* < 0.05). No significant differences in blood C3 concentrations were detected among treatments at any time point, and *E. coli* challenge did not alter C3 levels in either the control or TM groups (*p* > 0.05).

AGP is an acute-phase protein synthesized by the liver in response to inflammation or tissue injury. In poultry, elevated AGP serves as a sensitive biomarker of infection, stress, and gastrointestinal inflammation, reflecting its role in regulating immune responses and maintaining tissue homeostasis (54, 55). In the present study, *E. coli* challenge elicited a marked increase in circulating AGP levels in the control group, consistent with activation of an acute inflammatory response. In contrast, TM altered the immune response to *E. coli* such that a pronounced early systemic AGP response was not observed in TM-treated birds.

Although AGP alone cannot be used to determine whether an inflammatory response is beneficial or detrimental, the reduced early AGP induction in TM-treated birds, when interpreted alongside other findings in the present study, suggests attenuation of early systemic inflammatory signaling rather than complete suppression of host defense mechanisms. Specifically, TM was associated with coordinated alterations in innate immune signaling pathways and inflammatory mediators, including TLR-2, TLR-4, NF-κB/p65, iNOS, and TNF-α, as well as modulation of the immunoregulatory cytokine TGF-β. Together, these changes are consistent with a dampened early inflammatory tone following *E. coli* challenge.

Within this context, the limited early AGP induction observed in TM birds likely reflects a regulated attenuation of acute-phase inflammatory responsiveness, rather than an absence of inflammatory capacity. Acute inflammation remains essential for pathogen control; however, excessive or poorly regulated inflammatory responses can be detrimental. The present findings therefore suggest that TM may influence the magnitude and timing of early inflammatory responses following bacterial challenge. Importantly, the functional consequences of this attenuated inflammatory profile cannot be determined from AGP measurements alone and warrant further investigation incorporating bacteriological outcomes, clinical parameters, and longitudinal immune assessments.

C3 is a crucial molecule in complement activation that aids pathogen recognition, opsonization, and clearance, as well as connecting innate and adaptive immunity (56). A comprehensive understanding of 3′s structure and its interactions could potentially facilitate the development of novel therapeutic strategies for infectious diseases (56).

In this study, neither *E. coli* infection nor TM had a significant effect on total C3 levels. Consistent with our findings, Kim et al. (57) reported that intraperitoneal *E. coli* injection in mice did not alter circulating C3 concentrations. Nevertheless, they observed increased C3a and C3b, indicating that *E. coli* infection activates the complement system through the classical, alternative, and lectin pathways. The relatively stable C3 concentration is likely explained by compensatory upregulation of C3 synthesis in response to infection, which counterbalances its consumption. Similarly, the unchanged C3 levels observed in our study from days 1 to 5 post-infection suggest a dynamic equilibrium between complement activation and replenishment, maintained by the host's acute-phase response. However, since we did not directly measure C3a and C3b, the extent of complement activation could not be fully assessed. Future studies should quantify both intact C3 and its cleavage products to provide a more comprehensive understanding of these dynamics.

### 3.6 Effects of TM and post-hatch *E. coli* challenge on broiler chickens' IgA, IgM, and IgY serum levels

**Figure 6:**
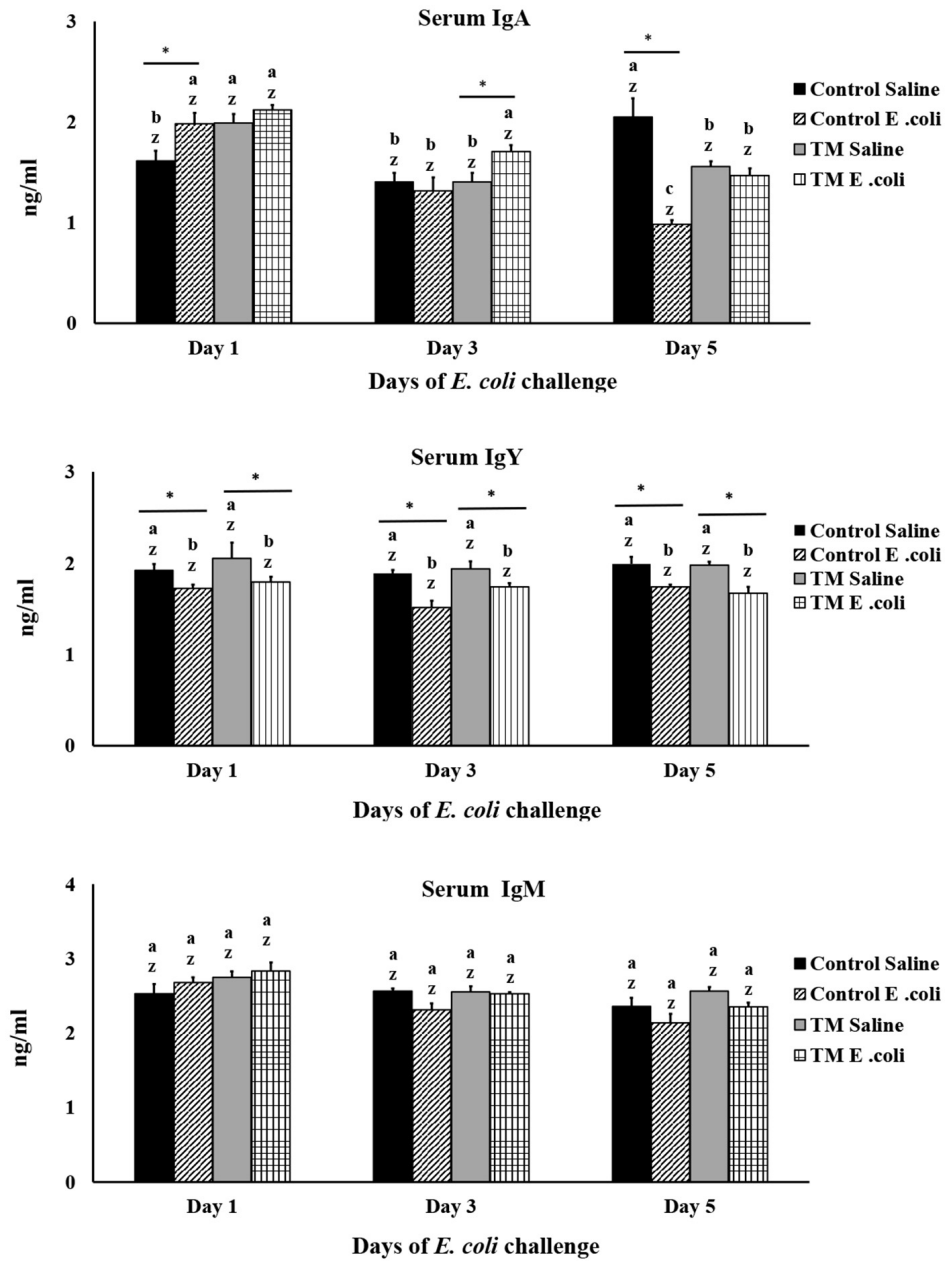
Effects of thermal manipulation (TM) during embryogenesis on the serum concentrations of IgA, IgM, and IgY in broiler chickens following *Escherichia coli* challenge. Means with different superscripts are statistically significantly different (*p* < 0.05). *,* Within the same day, the mean of the TM group differs significantly from that of the control group (*p* < 0.05). Error bars represent mean ± standard deviation (SD). Immunoglobulins represent total circulating Ig levels.

The effects of TM and *E. coli* challenge on total circulating serum IgA, IgM, and IgY levels in broiler chickens are presented in Figure 6. It should be emphasized that the immunoglobulins measured in this study represent total antibody levels rather than antigen-specific responses. As such, these measurements reflect the overall circulating immunoglobulin pool generated in response to multiple antigenic exposures, including diet, environment, commensal microbiota, and maternally derived antibodies, rather than *E. coli* –specific humoral immunity.

Within this framework, differences in immunoglobulin levels primarily reflect the influence of TM on systemic humoral immune regulation rather than direct effects of *E. coli* challenge on antibody production. TM was associated with altered temporal patterns of circulating IgA, characterized by a delayed but more sustained elevation during the early post-infection period (*p* < 0.05). However, given the short sampling window (1, 3, and 5 days post-challenge), these changes are unlikely to represent *de novo* antibody synthesis induced by *E. coli*. In chickens, primary IgM responses typically begin to rise several days after antigen exposure (approximately 3–7 days), whereas primary IgY and primary IgA responses generally occur later (7–10 days or beyond) (58). Therefore, the observed IgA dynamics likely reflect modulation, redistribution, or utilization of pre-existing antibody pools rather than pathogen-specific adaptive responses.

In contrast, total serum IgM levels were not significantly affected by TM or post-hatch *E. coli* challenge during the early sampling period (*p* > 0.05). Given the known kinetics of IgM production and its role in early adaptive immunity, the absence of detectable changes further supports the interpretation that the study time frame captured modulation of existing antibody pools rather than infection-induced antibody generation. In this study, *E. coli* reduced IgY levels in both groups after infection (*p* < 0.05). This decrease likely results from existing IgY antibodies in the serum, which are key components of the humoral immune system, quickly binding to these antigens and forming immune Complexes (antigen-antibody complexes).

Overall, these findings indicate that embryonic thermal manipulation influences the regulation and temporal dynamics of the total circulating IgA pool during early life. However, because antibody measurements were limited to early post-challenge time points and did not include antigen-specific assays or pre-challenge baselines, the data should not be interpreted as evidence of *E. coli*–specific humoral immunity. Future studies incorporating pre-challenge sampling, antigen-specific antibody measurements, and extended post-infection follow-up (e.g., 1–3 weeks) are required to determine how TM affects adaptive humoral immune responses to bacterial challenge.

## 4 Conclusion

The present study demonstrates that embryonic thermal manipulation modulates early immune-related parameters in broiler chickens following post-hatch *Escherichia coli* challenge. Specifically, thermal manipulation was associated with altered expression of innate immune signaling molecules and inflammatory mediators, as well as changes in acute-phase protein (α1-acid glycoprotein) responses and the temporal dynamics of total circulating immunoglobulins during the early post-infection period. Importantly, the observed differences in AGP and immunoglobulin levels should be interpreted as indicators of modified immune regulation rather than as evidence of enhanced disease resistance or pathogen-specific immunity. Given that immunoglobulin measurements reflected total antibody pools and sampling was limited to early post-challenge time points, the findings do not represent *de novo* adaptive immune responses. In addition, the experimental period extended only until day 25 post-hatch, and production performance, clinical outcomes, and bacteriological measures were not assessed. Future studies incorporating extended rearing periods to market age, antigen-specific immune assessments, and comprehensive performance and health parameters are therefore required to fully elucidate the role of embryonic thermal manipulation in poultry immune development and resilience.

## Data Availability

The real-time qPCR run files and raw data are available on GitHub at https://github.com/mbalzghoul/Thermal-manipulation-and-immune-response.
